# Antifouling Modification of Gold Surfaces for Acoustic Wave Sensor Applications

**DOI:** 10.3390/bios15060343

**Published:** 2025-05-29

**Authors:** Aries Delica, Mikhail A. Nazarov, Brian De La Franier, Michael Thompson

**Affiliations:** Department of Chemistry, University of Toronto, 80 St. George Street, Toronto, ON M5S 3H6, Canada; aries.delica@mail.utoronto.ca (A.D.); mikhail.nazarov@mail.utoronto.ca (M.A.N.); brian.delafranier@mail.utoronto.ca (B.D.L.F.)

**Keywords:** surface-modified gold, gold-based biosensor, acoustic wave biosensor, non-specific adsorption, antifouling coating

## Abstract

This study aims to develop a robust and reproducible method for fabricating efficient ultrathin antifouling coatings on gold surfaces by leveraging hydroxylation-based surface modifications. An ultrathin antifouling coating of a monoethylene glycol silane derivative, known to reduce fouling by at least 90% on flat hydroxylated surfaces, was successfully replicated on flat gold (reducing fouling by ~75%) by hydroxylating its surface with β-mercaptoethanol. This tandem coating contains the monoethylene glycol silane layer on top of the β-mercaptoethanol on the gold. Characterization was performed using contact angle goniometry, atomic force microscopy, x-ray photoelectron spectroscopy, and antifouling measurements. The results from these techniques, consistent with the literature, confirmed the successful and reproducible application of the tandem coating. Through heterogeneities, including defects and incomplete coverage, the AFM data revealed distinct visible layers of the tandem coating. The direct application of monoethylene glycol silane onto gold resulted in superior antifouling performance (88% reduction), demonstrating that direct silylation exploits pre-existing oxygen-containing species on the gold surface for a more effective antifouling layer. These findings offer a scalable approach for engineering antifouling coatings on gold substrates, with potential applications in biosensing and implantable device antifouling technologies.

## 1. Introduction

Gold surface-based sensors are ubiquitous in the analytical and clinical community as they are often used as biosensors to detect biomarkers that are indicative of diseases such as cancers or pathogens. Some examples of these surface-based sensors can be seen in quartz crystal microbalances, surface plasmon resonance devices, or even in simple antibody assays [1,2,3]. These devices generally function by employing a biorecognition element or receptor on the surface of the sensor that is sensitive to the biomarker of interest, where a biomarker–biorecognition interaction produces a measurable signal. However, one major barrier to effective sensing of these biomarkers is fouling (also known as non-specific adsorption), which can adversely affect the sensitivity and selectivity of the sensor [4]. This prevents the practical analysis of real-world samples such as whole blood or serum, due to the significant loss of sensitivity and selectivity. The importance of developing effective antifouling coatings also has useful implications other than in biosensing, such as in medical implants and devices. Thus, discovering effective antifouling coatings that can be utilized to prevent bacterial adhesion/infection, clotting, or in immune responses in applications such as biosensors, catheters, or drug nanoparticles would be incredibly useful [5,6,7].

Consequently, one major area in the literature of gold-based biosensors focuses on the development of antifouling technologies, with self-assembled monolayer (SAM) coatings being a prevalent method to prevent fouling [8,9,10,11]. The SAM technique is often used for antifouling applications because of its ease of application on a surface where SAM molecules spontaneously assemble to form a monolayer film [12]. Additionally, these molecules can be modified to exhibit various surface properties for antifouling purposes [13]. Alternatively, grafted polymer coatings are commonly used as an antifouling coating as they offer denser coatings than SAMs and can interweave with each other, and are also tunable [14]. Generally, although polymer antifouling coatings can outperform SAM coatings [15], the implementation of SAMs is easier and more straightforward. Consequently, the ideal antifouling coating would combine both the ease of application with SAMs and the high antifouling performance of a polymer brush.

**Figure 1:**
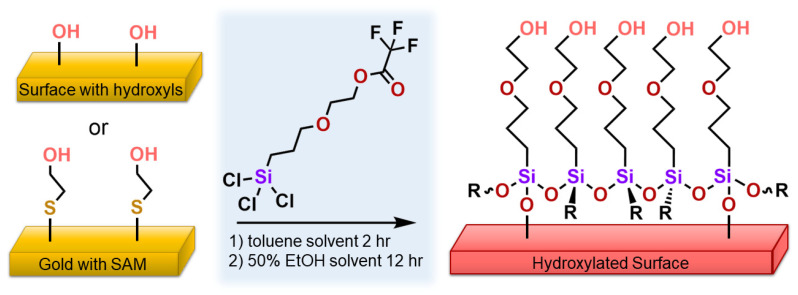
Concept of the Si-MEG-OH silica network formation on a naturally hydroxylated surface, such as silica quartz, or a gold surface modified with SAM-containing hydroxyls.

One notable coating is a monoethylene glycol silane named MEG-OH or Si-MEG-OH, which can reduce fouling of undiluted goat serum by up to 90% on a silica surface-based sensor [16,17]. This coating is an ultrathin silane polymer layer that consists of a covalent siloxane network and is formed from trichlorosilane molecules that undergo condensation reactions on a hydroxylated surface, such as a quartz disk (Figure 1) [18]. This antifouling coating inherits properties of SAMs and polymer brushes, as it is easy to employ on surfaces due to spontaneous self-assembly, but also has 3D polymer brush-like properties due to intermolecular cross-linking of silane groups [19]. This coating is not limited to quartz surfaces; it has been found to be effective on other hydroxylated surfaces, such as stainless steel stents against whole blood, or plasma-treated polyurethane against bacteria, with similar antifouling success [10,15]. However, previous applications of this antifouling layer using a SAM thiol version have not worked on gold. It is hypothesized that this is due to inadequate spacing between the monoethylene molecules in comparison with Si-MEG-OH [20,21]. Adequate spacing between the monoethylene backbones allows for an interstitial water layer to form, which acts as a hydrophilic barrier, preventing surface fouling [22]. Therefore, this unique SAM silane coating can potentially offer an antifouling performance comparable to polymer brushes, while also being easily employed on gold surfaces.

Hence, if it is possible to conserve the covalent siloxane network when applying Si-MEG-OH on gold, then it may be possible to obtain good antifouling results (up to 90% in fouling reduction) as seen in other previous Si-MEG-OH applications [6,11,16,17,18]. However, as gold does not have natural hydroxyls on its surface, it must be chemically modified to allow for the same trichlorosilane reactions to occur on the surface. One approach to hydroxylate gold is through the use of hydroxyl SAMs such as β-mercaptoethanol (βME), which is a well-known method to modify gold surfaces [23]. After coating with βME, the Si-MEG-OH precursor is reacted with the βME hydroxyls to form a tandem βME/Si-MEG-OH coating. To characterize this coating, contact angle goniometry and atomic force microscopy (AFM) are performed to provide evidence that a Si-MEG-OH coating can exist on gold. The antifouling performance of the tandem coating on gold is also tested with a thickness shear mode (TSM) sensor using modified gold electrodes on quartz disks. These disks can be exposed to undiluted goat serum and can be compared to previously existing fouling data of Si-MEG-OH (~90% fouling reduction). A comparison to gold disks that have been directly modified with Si-MEG-OH is also performed to determine if the βME coating is necessary. Furthermore, qualitative XPS measurements from the βME and Si-MEG-OH are performed to further confirm that these disks contain the expected coatings.

## 2. Materials and Methods

### 2.1. Sources of Materials

All the chemicals were purchased from Sigma-Aldrich, Oakville, ON, Canada, unless otherwise noted. The TSM gold–quartz crystals (AT-cut 13.5 mm diameter, 9 MHz) and quartz crystals (AT-cut, 13 mm diameter, 83 μm thickness, 20 MHz frequency) were purchased from Lap-Tech Inc., Bowmanville, ON, Canada. Silicon Wafers (Ultra-Flat, 6″, Prime/CZ Virgin, P/Boron doped, <100>, 1–30 Ohm/cm, Thickness: 650 ± 10 µm) were purchased from Alpha Nanotech, Vancouver, BC, Canada. Gold-coated substrates (250 ± 50 nm gold on 1–4 nm chromium on borosilicate glass base) were purchased from Arrandee, Werther, Germany. Goat serum (Gibco brand, New Zealand origin) was purchased from Thermo Fisher Scientific Inc., Mississauga, ON, Canada.

### 2.2. Synthesis and Characterization of 2-(3-Trichlorosilylpropyloxy)-ethyltrifluoroacetate

The Si-MEG-OH precursor, 2-(3-trichlorosilylpropyloxy)-ethyltrifluoroacetate (Si-MEG-TFA), was synthesized using previously described methods from De La Franier et al. [16] by reacting 2-(allyloxy)ethyl 2,2,2-trifluoroacetate (1.0 equiv., synthesized) with H_2_PtCl_6_•6H_2_O (1.0 mol%, reagent grade) and HSiCl_3_ (2 equiv., 99%) in an inert atmosphere nitrogen glovebox (N_2_, Linde, 4.8 Industrial Grade, 99.998%) for 20 h via Pt-catalyzed hydrosilylation. The final product was isolated by distillation under vacuum and was characterized by 1-D and 2-D ^1^H and ^13^C NMR in CDCl_3_ using a Bruker Ascend 400 NMR Spectrometer (Bruker BioSpin GmbH, Ettlingen, Germany).

### 2.3. Cleaning and Coating Procedures

The cleaning and coating procedures were derived from previous methods [22]. The gold substrates were cleaned by being placed in test tubes, followed by rinsing with distilled water three times, rinsing with 1% sodium dodecyl sulfate (SDS) solution twice, followed by strong orbital shaking in SDS solution for 15 min. The substrates were rinsed with acetone three times and then rinsed with methanol twice, followed by strong orbital shaking in methanol for 5 min. The substrates were dried under a stream of nitrogen and were plasma-cleaned under vacuum in ambient air (Harrick PDC-3XG, Ithaca, NY, USA) for 5 min.

To coat the substrates with βME, clean substrates were placed in test tubes and were submerged in 95% ethanol solution, and approximately 0.5% *v*/*v* of βME was added. The solution was placed on an orbital shaker for a minimum of 2 h before rinsing with methanol and drying with a stream of nitrogen gas.

To coat bare gold or gold modified with βME with Si-MEG-OH, the substrates were placed in test tubes that had been dried in a 180 °C oven for at least 90 min, then placed in a humidity chamber, maintained at 70% relative humidity with a saturated aqueous solution of MgNO_3_•6 H_2_O. The substrates were then transferred to a nitrogen glovebox, submerged in anhydrous toluene with approximately 1% *v*/*v* of Si-MEG-TFA added. The test tubes were sealed in nitrogen using rubber stoppers and were placed on an orbital shaker for at least 2 h. The test tubes were then unsealed, and the substrates were thoroughly rinsed with toluene three times, then thoroughly rinsed with 95% ethanol three times. The substrates were finally submerged in 50% ethanol to convert the Si-MEG-TFA terminal group into Si-MEG-OH and were placed on an orbital shaker for at least 12 h before rising with methanol, drying with nitrogen, and storing in glass scintillation vials.

For the substrates that were directly modified with Si-MEG-OH, the above salinization procedure was used, except that the β-mercaptoethanol treatment step was skipped. These direct Si-MEG-OH treatments were applied to the bare gold, quartz crystals, and silicon wafer samples.

### 2.4. Characterization of the Modified Gold and Silicon-Based Substrates

To characterize the coatings on gold, contact angle goniometry and AFM measurements were performed to assess hydrophobicity and changes in surface morphology, respectively, which provides evidence of the successful application of βME and/or Si-MEG-OH layers. The coatings of βME and Si-MEG-OH were also analyzed on silica quartz and bare silicon substrates as a basis for comparison. To determine the antifouling performance of the tandem βME/Si-MEG-OH and direct Si-MEG-OH coatings on gold, TSM gold electrodes on quartz disks were modified with the tandem coating and were exposed to undiluted goat serum, to be compared to a bare gold treatment and referenced to the literature. XPS measurements were also performed on the gold TSM disks for each treatment step to confirm the presence of the expected coatings.

#### 2.4.1. Contact Angle Measurements

Contact angle goniometry was measured and analyzed using a KSV CAM 101 and its software (KSV Instruments Ltd., Helsinki, Finland) using 10 µL of distilled water for each gold substrate. Triplicate samples were measured for each coating condition.

#### 2.4.2. AFM Surface Characterization

Surface characterization of the substrates was performed using an AFM with peak force tapping mode (AFM Inspire with Peak Force IR and Nanoscope V, Bruker, Germany). The images were recorded in Peak Force—AFM mode with a peak force tapping frequency of 2 kHz, peak force amplitude of 30 nm, and an applied force of 3 nN at a scan rate of 0.2 Hz using a Pt-coated cantilever with a spring constant of 5 N/m (HQ:NSC14/Pt, MikroMasch, Watsonville, CA, USA) unless stated otherwise. The spring constant and the resonant frequency were measured via the thermal tune method. The captured maps were saved as “.spm” files and opened in the software, Gwyddion (Version 2.62), for further basic artifacts correction and data analysis.

#### 2.4.3. TSM Antifouling Measurements

**Scheme 1:**
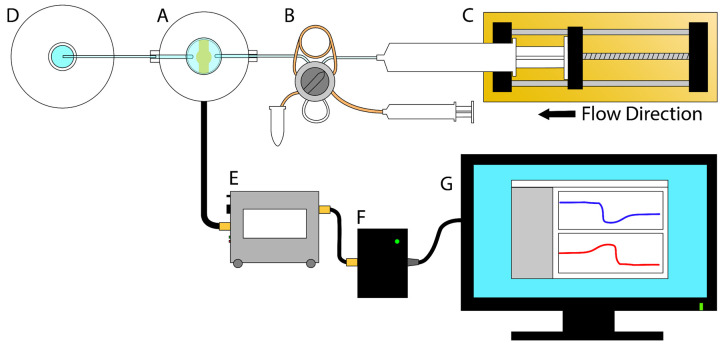
General schematic of the complete TSM setup for experimentation and data collection, including (**A**) flow chamber with TSM crystal, (**B**) injection loop, (**C**) syringe pump, (**D**) waste bottle, (**E**) PLO-10i oscillator, (**F**) A/D convertor, and (**G**) computer interface.

Antifouling tests of the TSM disks were performed using a procedure adapted from previous MEG-OH TSM experiments (Scheme 1) [22]. A syringe pump holding standard PBS solution was operated in push mode with a flow rate of 90 µL/min, connected to a loop injector with a sample loop capacity of 250 µL, and filled with undiluted goat serum. This was connected to a custom TSM disk cell holder, which allowed for one side of the disk to be exposed to solution and for an electrical connection with a Maxtek PLO-10i oscillator (Inficon, East Syracuse, NY, USA). Analog signals from the oscillator were converted into recordable digital data using a custom-designed analog-to-digital circuit using a Parallax QuickStart board (Parallax, Rocklin, CA, USA), and custom software created in Visual Basic was used to collect and record real-time frequency data. The experiments were run by first conditioning the TSM disk with 95% ethanol, then using the syringe pump to flow PBS solution until a steady-state equilibrium of the disk frequency was reached. The loop injector was then used to expose the disk to goat serum until another steady-state was established.

#### 2.4.4. XPS Analysis of the TSM Crystals

XPS measurements with a take-off angle of 45˚ were performed with a Theta probe ThermoFisher Scientific Instrument (East Grinstead, UK). Modified TSM gold electrodes on quartz crystals with the bare, βME, βME/Si-MEG-OH, and direct Si-MEG-OH treatments were analyzed with monochromated Al Kα X-rays. Spectra were fitted using the Advantage (Version 6.9.0, Build 00059) software by Gaussian/Lorentzian convolution functions and were referenced to adventitious C1s at 284.8 eV.

## 3. Results and Discussion

### 3.1. NMR Characterization of Si-MEG-TFA

The NMR analysis (reported in S1 in the Supplementary Data) showed that Si-MEG-TFA was successfully synthesized, as the 1-D ^1^H signals are consistent with previous data [16], and that the 2-D NMR spectra are consistent with the proposed structure.

### 3.2. Contact Angle Measurement Results

**Figure 2:**
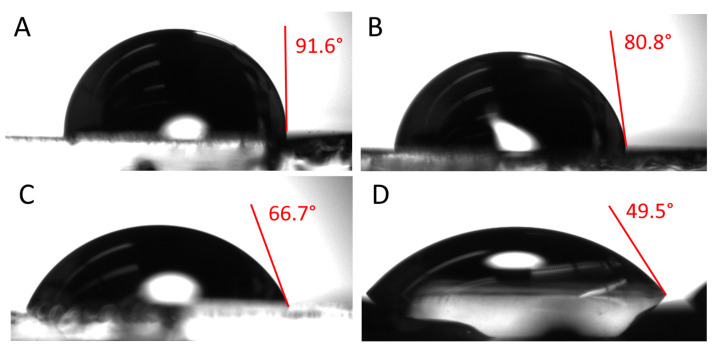
Contact angle images of water on gold-coated silica substrates when bare (**A**), coated with βME (**B**), then subsequently coated with Si-MEG-TFA (**C**), and coated with Si-MEG-TFA directly without βME (**D**).

The bare gold substrate showed a contact angle of 91.6 ± 1.2° (Figure 2). After treating with βME, the contact angle decreased to 80.8 ± 1.6°, which further decreased to 66.7 ± 2.3° after coating with Si-MEG-OH on top of the βME. Additionally, directly coating the gold with Si-MEG-TFA resulted in the lowest contact angle at 49.5 ± 0.6°. These trends in decreasing contact angle are consistent with contact angles of Si-MEG-OH on other surfaces such as stainless steel or silica quartz [3,4], suggesting that the Si-MEG-OH was successfully applied to the gold’s surface. Furthermore, the contact angles for the gold Si-MEG-OH coatings are higher than expected from the literature, where contact angles around 30° for Si-MEG-OH coatings are observed [24,25,26,27]. This implies that the bare surface was not completely free of contaminants, as these other studies were able to obtain lower contact angles of bare gold (down to ~48–66°) when cleaned by other methods, such as basic piranha solution [27,28]. Therefore, it may be possible to achieve lower contact angles and better antifouling results on cleaner bare gold surfaces.

### 3.3. AFM Results of Modified Substrates

**Figure 3:**
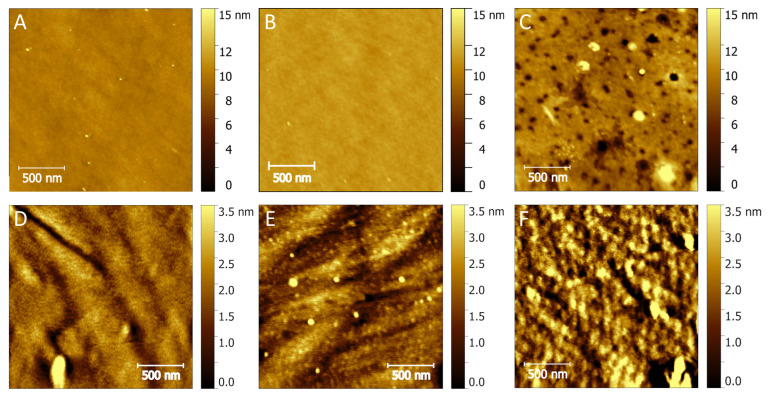
AFM images of silicon substrates when bare (**A**), then coated with βME only (**B**), then subsequently coated with Si-MEG-OH (**C**), and of silica quartz crystal substrates when bare (**D**), then coated with βME only (**E**), then subsequently coated with Si-MEG-OH (**F**). 2 µm scales.

To confirm that the substrates were successfully coated, their morphologies were characterized by AFM. To serve as a comparison for the gold substrates and their treatments, AFM images of the pure silicon substrate and silica quartz substrate under different conditions (bare, βME-coated, or a tandem βME/Si-MEG-OH-coated) were recorded (Figure 3). Both the silicon and silica substrates have significant morphological changes when comparing the Si-MEG-OH treatments to their bare counterparts. For the silicon substrate, the success of the tandem coating is evident with notable holes/voids in the tandem contrasted with a smoother surface in the bare coating (Figure 3A,C). The Si-MEG-OH coating can be described as a solid film with a thickness of 25 nm with frequent depressions of 50–100 nm in size and a depth of 10 nm (Figure 3C). Similarly, in the silica quartz substrate, the morphology of the tandem coating Si-MEG-OH is rougher, contains holes, and no longer contains boundary lines relative to bare silica (Figure 3D,F). Both AFM comparisons of the bare and Si-MEG-OH coating support successful Si-MEG-OH coverage due to their morphological differences. These differences are also consistent with differences in contact angles of bare and Si-MEG-OH silica-based substrates in the literature [16,24], as changes in the surface morphology also correspond to changes in the substrate’s contact angles. Additionally, it was found that the application of βME, which is expected to coat only gold surfaces, appears to coat silica quartz (Figure 3E), as evidenced by a distinct periodic spotting pattern of objects that are 60 nm in diameter and 1–5 nm high, which are absent on the clean planes of the empty quartz substrate. This pattern is indicative of a fully coated SAM layer when compared to the literature [29,30,31].

**Figure 4:**
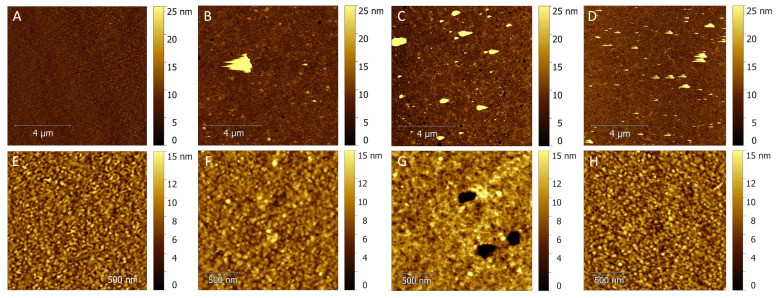
AFM images of gold-coated silica substrates when bare, then coated with βME, then subsequently coated with Si-MEG-OH, and coated with Si-MEG-OH directly without βME. 10 µm scale (**A**–**D**) and 2 µm scale (**E**–**H**).

AFM images of the gold substrates were recorded for the bare, βME coating, tandem βME/Si-MEG-OH coating, and direct Si-MEG-OH coating (Figure 4). Large aggregates were observed as spheroidal tall objects on all the treatments except for the bare, which could represent nanoparticles or precipitates (Figure 4B–D) [32]. The βME on gold (Figure 4B,F) is similar to the βME on silica quartz, as the same periodic spotting pattern is seen again on the surface, supporting a successful coating of βME. However, the large difference in roughness between the gold (~10 nm) and silica substrates (~2.5 nm) makes it especially difficult to determine and confirm the βME coating on the gold substrate due to its initial bare surface morphology. Additionally, holes/voids 15–20 nm deep and ~300 nm in diameter were observed in all the coatings (Figure 4B,C,G) but not observed in the bare treatment, implying that the treated surfaces are predominantly coated but have imperfections. The presence of holes/voids on other gold surfaces covered with SAMs is also reported in the literature, providing evidence of successful coating [33,34].

The necessity of the βME deposition step to obtain the full βME/Si-MEG-OH tandem coating is evident when comparing it with the direct Si-MEG-OH coating on the gold substrate (Figure 4G,H). The morphology of the direct coating (Figure 4H) is very similar to the bare gold substrate (Figure 4E), whereas the tandem coating (Figure 4G) is present as a fairly uniform layer with a roughness of up to 1 nm (Table S1), a completely different morphology.

**Figure 5:**
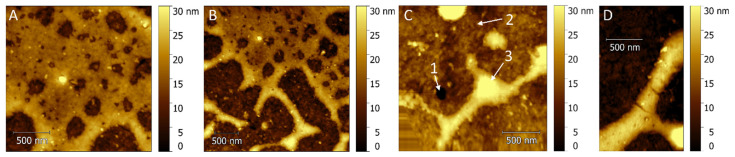
AFM images of the incomplete coating areas of the gold–silicon substrate with the full βME/Si-MEG-OH coating revealing distinct layers (**A**,**B**), holes on the bottom layer (**C**), showing bare gold (1) βME coating, (2) Si-MEG-OH, and (3) cracks on the apparent Si-MEG-OH layer (**D**).

Furthermore, areas of incomplete coverage were found in the tandem coating on the gold (Figure 5), which reveals distinct layers of the βME and Si-MEG-OH. This is evident since the top layer with the higher depth profile most likely represents the Si-MEG-OH coating, as it is consistent with the morphology of both gold and silicon coated with Si-MEG-OH (Figure 3C and Figure 4G). The bottom layer with the lower depth profile conversely represents the βME layer, as the morphology is more similar to the βME treatment (Figure 4B,F) than the bare treatment (Figure 4E) and is similar to the morphology of SAM thiol coatings on gold in the literature [33,34]. Moreover, it is shown that the bottom layer contains holes seen previously in the coating on the gold substrate (Figure 4B,C,G), which were never seen in the bare treatment. The presence of these holes (Figure 5C) serves as additional evidence of the bottom layer, most likely corresponding to the βME coating. Therefore, AFM Figure 5C represents all three layers in one image. This allowed us to establish that the thickness of the βME coating on gold is ~20 nm, while the thickness of the unknown Si-MEG-OH coating can vary from 15 to 40 nm. More detailed analyses are available in SI, Figure S10. Additionally, it was found that there were fault lines or cracks in the apparent Si-MEG-OH layer (Figure 5D), which may be indicative of an amorphous structure of Si-MEG-OH.

### 3.4. TSM Fouling Data with Undiluted Goat Serum

**Figure 6:**
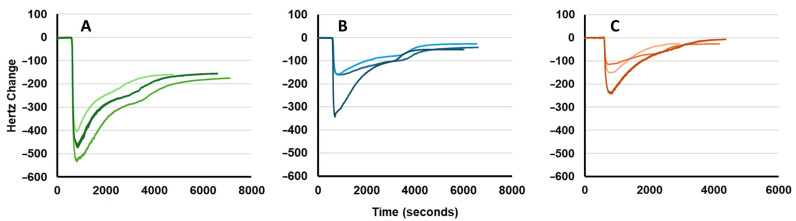
Triplicate TSM runs of bare gold (**A**), βME with Si-MEG-OH (**B**) against undiluted goat serum, and direct MEG-OH (**C**). Different shades are used for clarity to distinguish between trials in the figure.

**Figure 7:**
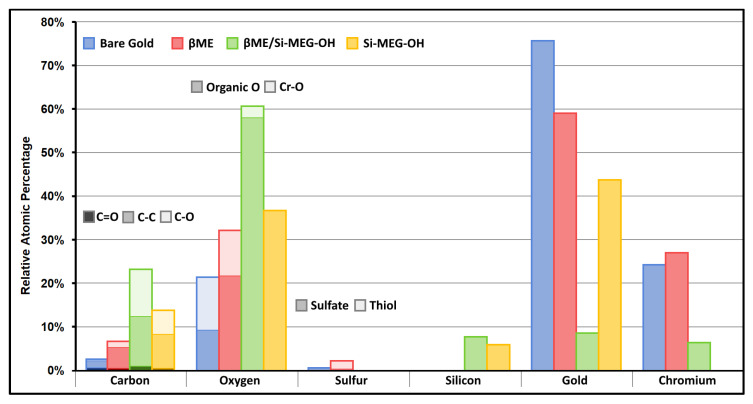
Relative atomic percentages of carbon, oxygen, sulfur, silicon, and gold for bare (blue), βME-coated (red), βME/Si-MEG-OH-coated (green), and gold directly coated with Si-MEG-OH (yellow) on the TSM gold–quartz crystals, calculated from Qualitative XPS analysis.

From the TSM measurements, it was found that the βME/Si-MEG-OH coating could reduce fouling by approximately 75% for the undiluted goat serum (Table 1 and Figure 6). In comparison to the literature, the Si-MEG-OH on pure quartz crystals resisted fouling by up to 90% [17,24], while the thiolated SAM version of MEG-OH on gold was found to have a frequency change of at least 100 Hz for 50% diluted goat serum using the same gold disk crystals [18]. These results highlight the importance of conserving the polymer siloxane network in Si-MEG-OH, as it outperforms its thiolated SAM version. It also suggests that further optimization of the tandem layer could result in better antifouling performance, due to the observed imperfections in the coating procedure, which is evident from the higher contact angles and large aggregates found in the AFM scans. Interestingly, directly coating the gold electrodes with Si-MEG-OH produced an even better antifouling result compared to the tandem coating, as it reduced fouling by 88% compared to bare crystal. These results are comparable to the performance of Si-MEG-OH coatings on quartz against serum in the literature [13]. Although the silane layer should not be able to bind to pure gold, there is a considerable amount of oxygen on the bare gold electrode, according to XPS data, to which the silane could be binding (Figure 7).

### 3.5. XPS Data for the TSM Gold Electrodes on Quartz Crystals

**Figure 8:**
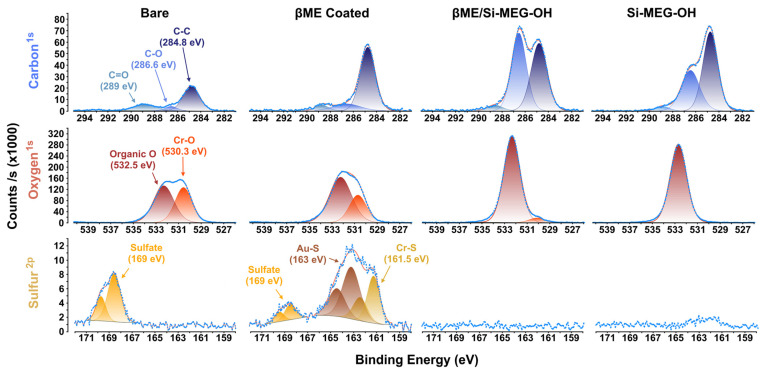
Qualitative XPS scans of the bare, βME-coated, βME/Si-MEG-OH-coated, and Si-MEG-OH-coated TSM gold electrodes on quartz crystals. Carbon^1s^ (**top** row), O^1s^ (**middle** row), and S^2p^ (**bottom** row) are shown.

XPS analysis of the TSM gold–quartz crystals (Figure 7, and C1-4) shows that there is (adventitious) carbon, oxygen, and sulfur contamination on the bare gold treatment. The chromium bonding layer for the gold is also visible in the XPS spectra. The majority of the contamination appears to be oxygen, which accounts for 20% of the relative atomic make-up of the surface, while gold accounts for 77%. This oxygen contamination shows two near equally intense peaks: one at 530 eV, attributable to chromium oxide [35], and one at 533 eV, attributable to organic oxygen [36] (Figure 8). Carbon and sulfur make up the rest of the contamination.

The successful coating of βME is evident with the presence of a gold thiol S^2p^ signal around 163 eV [37] in the βME spectra, compared to the sulfate contamination of gold at 169 eV (Figure 8). This thiol peak also shows a shoulder at 161 eV, likely attributable to a chromium thiol bond [34]. The relative amount of sulfur for the βME coating increased to 2% compared to less than 1% for the bare gold, and the carbon signal also increased on the addition of βME, lending further evidence that the coating was successfully applied. The carbon for the βME coating also showed a change in peaks compared to the bare gold, with the major peak at 285 eV (C-C bonds) dwarfing the smaller peaks at 287 eV (C-O) and 289 eV (C=O) [38]. This is expected, as βME has more C-C bonds in its internal structure compared to C-O bonds.

On addition of Si-MEG-OH to the βME coating, the sulfur signals completely disappear, suggesting it has been buried beneath the MEG-OH layer. The C-O peak at 287 eV is much stronger in the carbon scan on the addition of Si-MEG-OH, compared to the βME coating. At the same time, the oxygen scan predominantly shows a large peak at 532.5 eV, corresponding to organic oxygen, on addition of Si-MEG-OH, whereas for the bare and βME-coated gold, the chromium oxide peak at 530 eV is of comparable size. Therefore, C-O bonding changes in both carbon and oxygen spectra suggest that the Si-MEG-OH coating applied to the βME has become thick enough to almost entirely cover the metal oxide contamination of the bare gold. It is also evident that the Si-MEG-OH coating was successfully applied due to the appearance of a Si^2p^ signal relative to the bare and βME treatments, which did not show any peaks in this range.

When Si-MEG-OH is directly applied to gold, a Si^2p^ signal appears. The carbon and oxygen signal intensities greatly increase compared to the bare gold, but not as much as in the case of the βME/Si-MEG-OH coating (Figure 7). This confirms our hypothesis of the successful application of Si-MEG-OH directly to the gold surface, with a thinner coating than the βME/Si-MEG-OH dual layer. The direct Si-MEG-OH coating shows a higher ratio of C-C to C-O bonds compared to the βME/Si-MEG-OH coating, as seen by the larger peak at 285 eV compared to 287 eV (Figure 8). The overall structure of the βME/Si-MEG-OH coating has a similar ratio of C-C to C-O bonds as the direct Si-MEG-OH coating. Thus, the change in peak ratio observed is likely due to the thinner nature of the direct Si-MEG-OH, allowing more of the adventitious surface carbon to be visible.

## 4. Conclusions

Si-MEG-TFA, the Si-MEG-OH precursor, and the synthesis and isolation methodology were described. Its efficacy was confirmed by 1-D and 2-D NMR, and its key signals were consistent with the literature data. A pattern of decreasing contact angle of the tandem coating on gold substrate was shown to be consistent with other literature values of Si-MEG-OH coated on other hydroxylated surfaces. Furthermore, AFM analysis showed evidence of a successful βME layer deposition. Its morphology can be described as periodic spotting on the surface with the presence of holes, which is consistent with other SAM coating patterns from the literature. The Si-MEG-OH layer was found to be comparable to Si-MEG-OH on silica, and the presence of the tandem coating was evident by the observation of a double layer in areas of incomplete coverage, which had a top layer with a morphological pattern consistent with Si-MEG-OH and a bottom layer similar to βME.

TSM antifouling tests with βME/Si-MEG-OH gold electrodes on quartz crystals showed a significant reduction in fouling by approximately 75%, which is less but comparable to other antifouling applications of Si-MEG-OH on other hydroxylated surfaces. Additionally, XPS analysis of these samples qualitatively supports the presence of a tandem βME/Si-MEG-OH antifouling coating, highlighted with the emergence of a sulfide S^2p^ signal representing the sulfur from the βME layer and a silica Si^2p^ signal representing the Si-MEG-OH layer. Additionally, it was found that coating the gold electrode on silica directly with Si-MEG-OH produced the best antifouling result of 88%, even though the morphology is not apparent and looks similar to gold.

Overall, it may be possible to achieve better antifouling results for this coating, as contaminants and imperfections were found on the modified gold. Additionally, the coating methods used to apply the Si-MEG-OH were adapted for coating pure silica quartz crystals, which implies that there may be optimal cleaning and coating procedures resulting in much better antifouling results. Furthermore, because imperfections such as holes were observed in the βME/Si-MEG-OH coating, it may be possible to exploit areas that were not coated with a biorecognition element to allow for the detection of a specific analyte of interest. In conclusion, it was found that this Si-MEG-OH SAM coating outperforms similar SAMs for TSM applications relative to the literature, while being straightforward to employ on gold biosensor surfaces.

## Figures and Tables

**Table 1:** TSM Data of Si-MEG-OH Relative to Bare Gold. *n* = 3.

Coating *	Frequency Change (Hz)	RSD (%)
Bare	163.5 ± 10.7	6.54
βME + Si-MEG-OH	39.7 ± 12.6	31.6
Si-MEG-OH	19.8 ± 10.7	53.9

* Treatments are significant when compared to bare using the unpaired *t*-test (*p* < 0.001).

## Data Availability

Data are available upon request from the corresponding author.

## Supplementary Materials

The following supporting information can be downloaded at https://www.mdpi.com/article/10.3390/bios15060343/s1, S1. Data collection and processing of 2-(3-trichlorosilylpropyloxy)-ethyltrifluoroacetate; Figure S1. Proposed structure of 2-(3-trichlorosilylpropyloxy) -ethyltrifluoroacetate (MEG-TFA); Figure S2. Full ^1^H NMR spectrum of MEG-TFA. ^1^H NMR (400 MHz, CDCl3); Figure S3. Full ^13^C NMR spectrum of MEG-TFA. ^13^C NMR (101 MHz, CDCl3); Figure S4. gCOSY45 spectrum of MEG-TFA; Figure S5. gHSQC spectrum of the MEG-TFA; S2. XPS Survey Spectra; Figure S6. XPS survey spectrum of bare gold; Figure S7. XPS survey spectrum of βME-coated gold; Figure S8. XPS survey spectrum of βME- and Si-MEG-OH-coated gold; Figure S9. XPS survey spectrum of Si-MEG-OH-coated gold; S3. Supporting AFM Data; Table S1. AFM roughness data; Figure S10. A. AFM image of the incomplete coating areas of the gold–silicon substrate with the full βME/Si-MEG-OH coating revealing distinct layers; B. Line profiles corresponding to the holes in the βME coating (1), incomplete Si-MEG-OH coating areas (2, 3).
