# Supplementary material for: Antifouling Modification of Gold Surfaces for Acoustic Wave Sensor Applications
Source: Biosensors (Basel). 2025 May 29;15(6):343. doi: 10.3390/bios15060343 (PMC12191305; doi:10.3390/bios15060343)
Supplement: Supplementary file 1 [file biosensors-15-00343-s001.zip › biosensors-3611573-supplementary.pdf]

Supplementary Material

Antifouling Modification of Gold Surfaces for Acoustic Wave Sensor Applications

Aries Delica †, Mikhail A. Nazarov †, Brian De La Franier † and Michael Thompson *

Department of Chemistry, University of Toronto, 80 St. George Street, Toronto, ON M5S 3H6, Canada; aries.delica@mail.utoronto.ca (A.D.); mikhail.nazarov@mail.utoronto.ca (M.A.N.); brian.delafranier@mail.utoronto.ca (B.D.L.F.)

* Correspondence: m.thompson@utoronto.ca; Tel.: +1-416-978-3575

† These authors contributed equally to this work.

S1. NMR Data Collection and Processing of 2-(3-trichlorosilylpropyloxy)-ethyltrifluoroacetate

The sample was dissolved in CDCl3 using a 3mm NMR tube, and all NMR spectra were acquired on a Bruker Ascend™ 400 NMR Spectrometer (v(1H) = 400.13 Hz; Bruker BioSpin GmbH, Germany) equipped with a Z163739_0774 (PI HR-BBO400S1-BBF/H/D-5.0-Z SP) probe. 1D 1H spectra were acquired using a zgpg30 pulse sequence at 25.25 ºC, over a 6250 Hz spectral window with 65536 points, a 1.0000 s recycle delay, and 16 scans. 1D 13C spectra were acquired using a zgpg30 pulse sequence at 25.45 ºC, over a 28809.5 Hz spectral window with 131072 points, a 0.2000 s recycle de-lay, and 512 scans. 2D 1H/1H gCOSY45 spectra were acquired using a cosygpppqf_20230621_DCB pulse sequence at 25.45 ºC, over a 6250 Hz spectral window with 2048 (F2) by 256 points (F1), a 1.0000 s recycle delay, and 1 scan. 2D 1H/13C gHSQC spectra were acquired using a hsqcedetgpsisp2.3 pulse sequence at 25.55 ºC, over a 6250.0 Hz 1H and 20627.2 Hz 13C spectral window with 1024 (F2) by 256 (F1) points, a 1.0000 s recycle delay, and 4 scans. The spectra were processed using MestreNova software (v 14.3.3-33362, Santiago de Compostela, Research S.L., Spain).

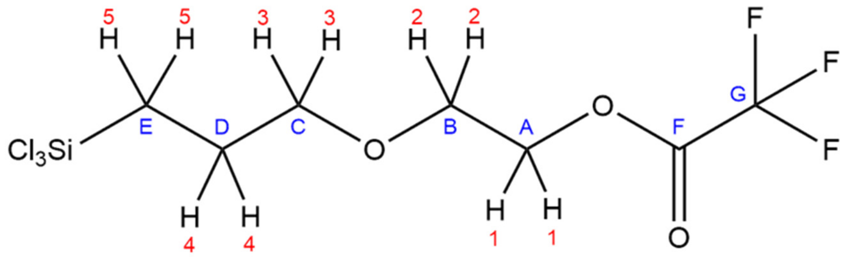

Figure S1. Proposed structure of 2-(3-trichlorosilylpropyloxy)-ethyltrifluoroacetate (MEG-TFA).

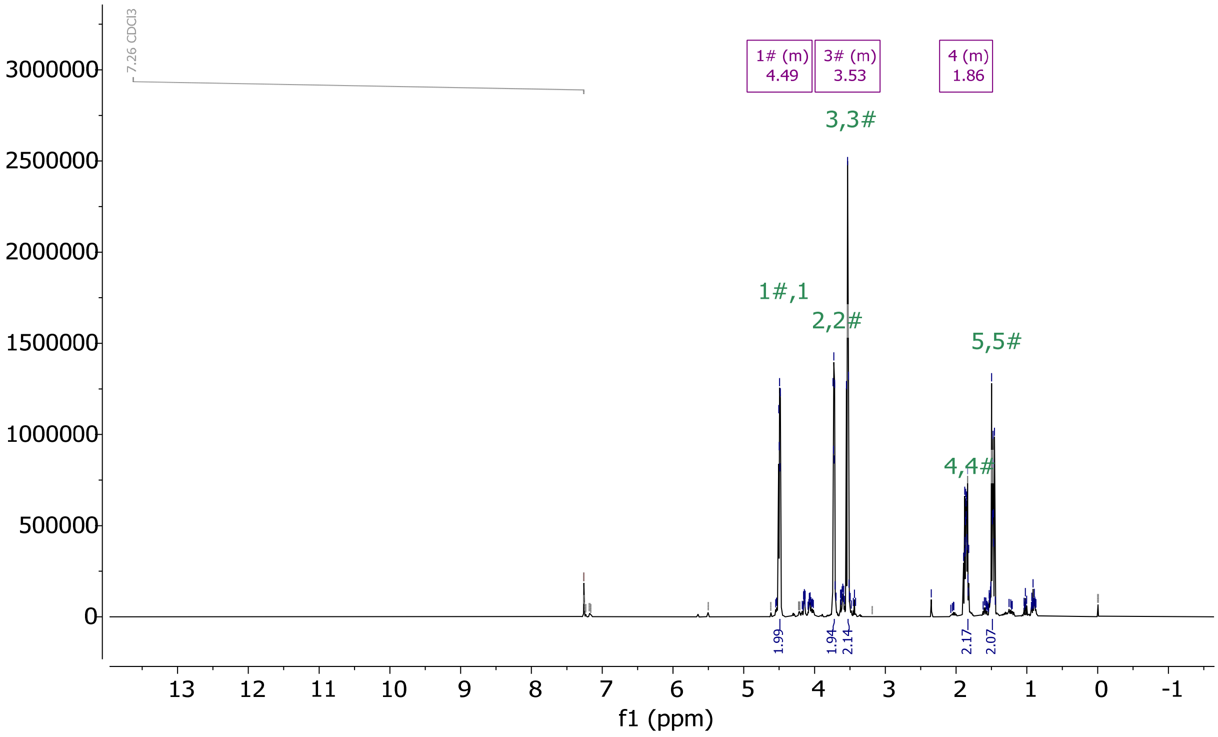

Figure S2. Full ^{1}H NMR spectrum of MEG-TFA. 1H NMR (400 MHz, CDCl3) δ 4.52 – 4.47 (m, 2H, H-1), 3.77 – 3.67 (m, 2H, H-2), 3.57 – 3.49 (m, 2H, H-3), 1.92 – 1.83 (m, 2H, H-4), 1.54 – 1.41 (m, 2H, H-5).

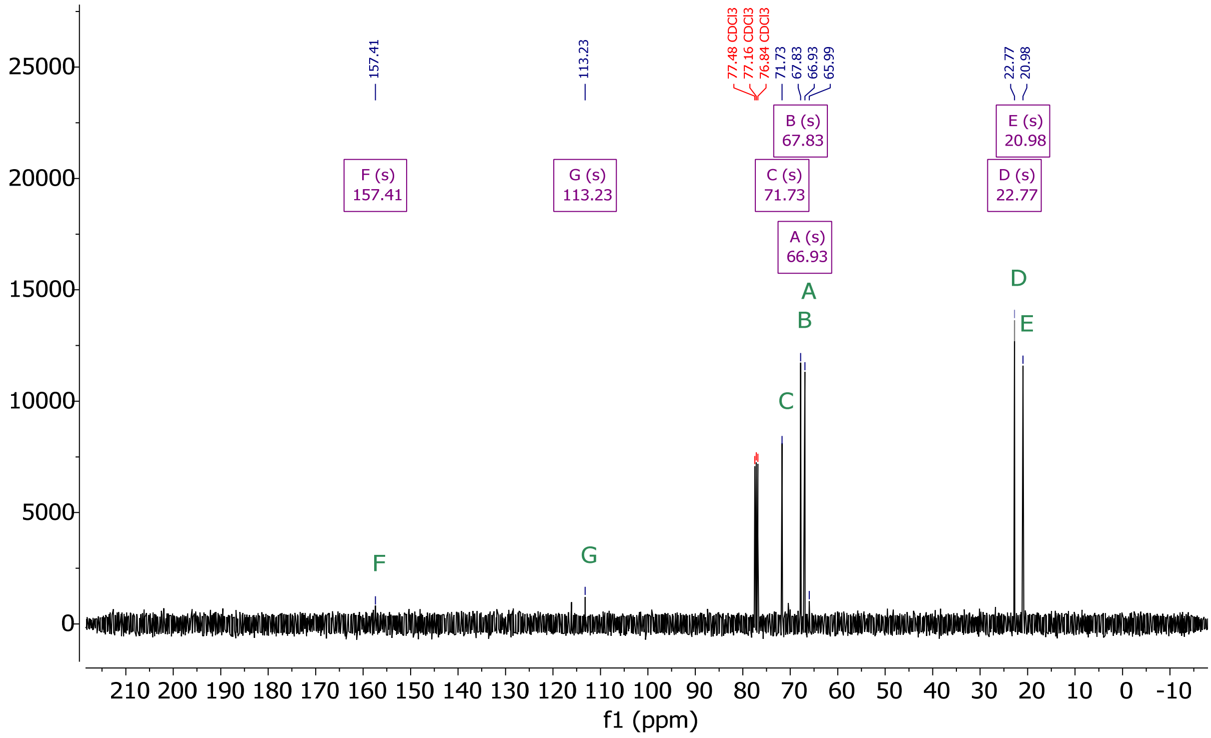

Figure S3. Full ^{13}C NMR spectrum of MEG-TFA. 13C NMR (101 MHz, CDCl3) δ 157.41 (C-F), 113.23 (C-G), 71.73 (C-C), 67.83 (C-B), 66.93 (C-H), 22.77 (C-D), 20.98 (C-E).

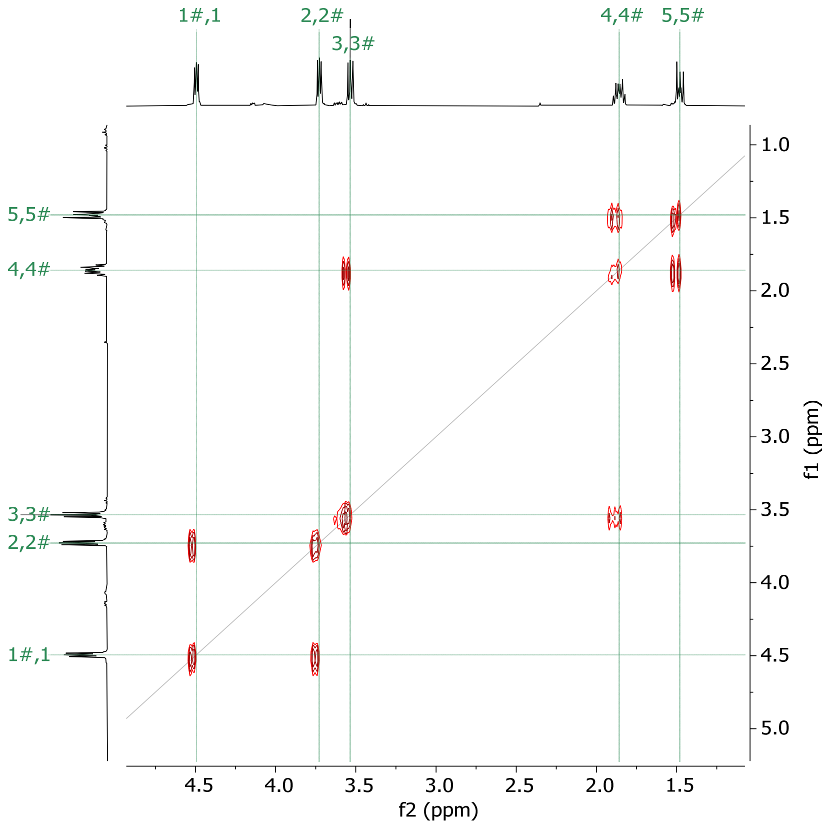

Figure S4. gCOSY45 spectrum of MEG-TFA.

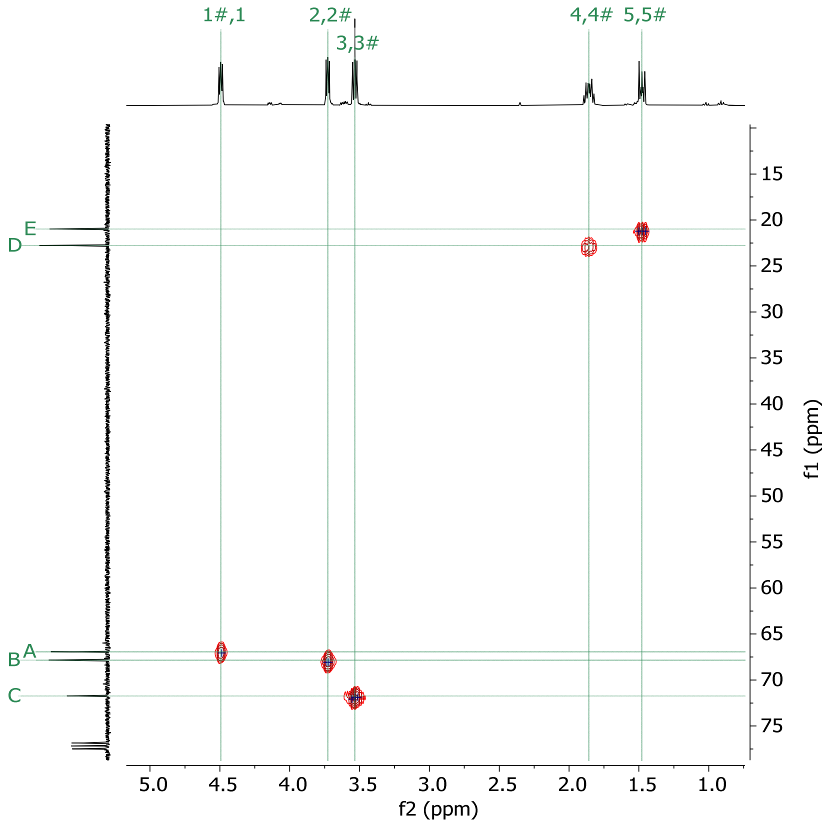

Figure S5. gHSQC spectrum of MEG-TFA.

S2. XPS Survey Spectra

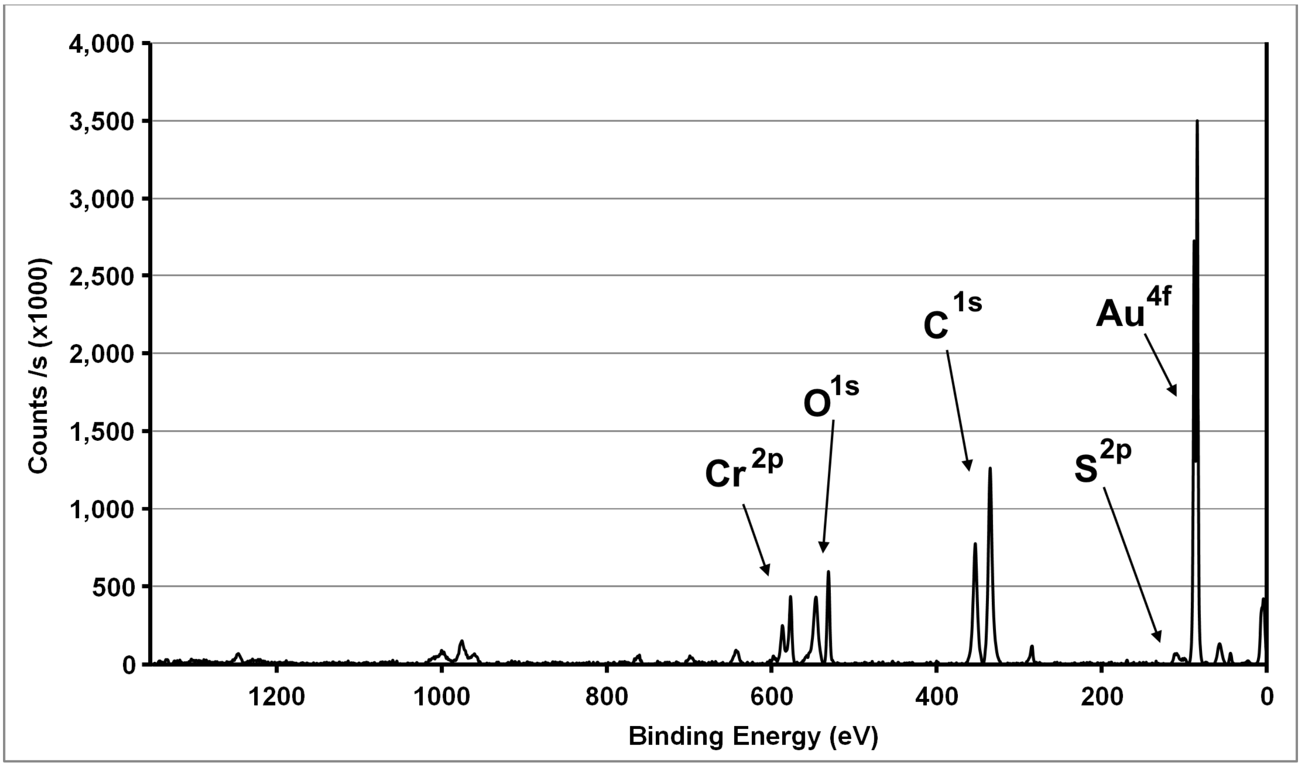

Figure S6. XPS survey spectrum of bare gold.

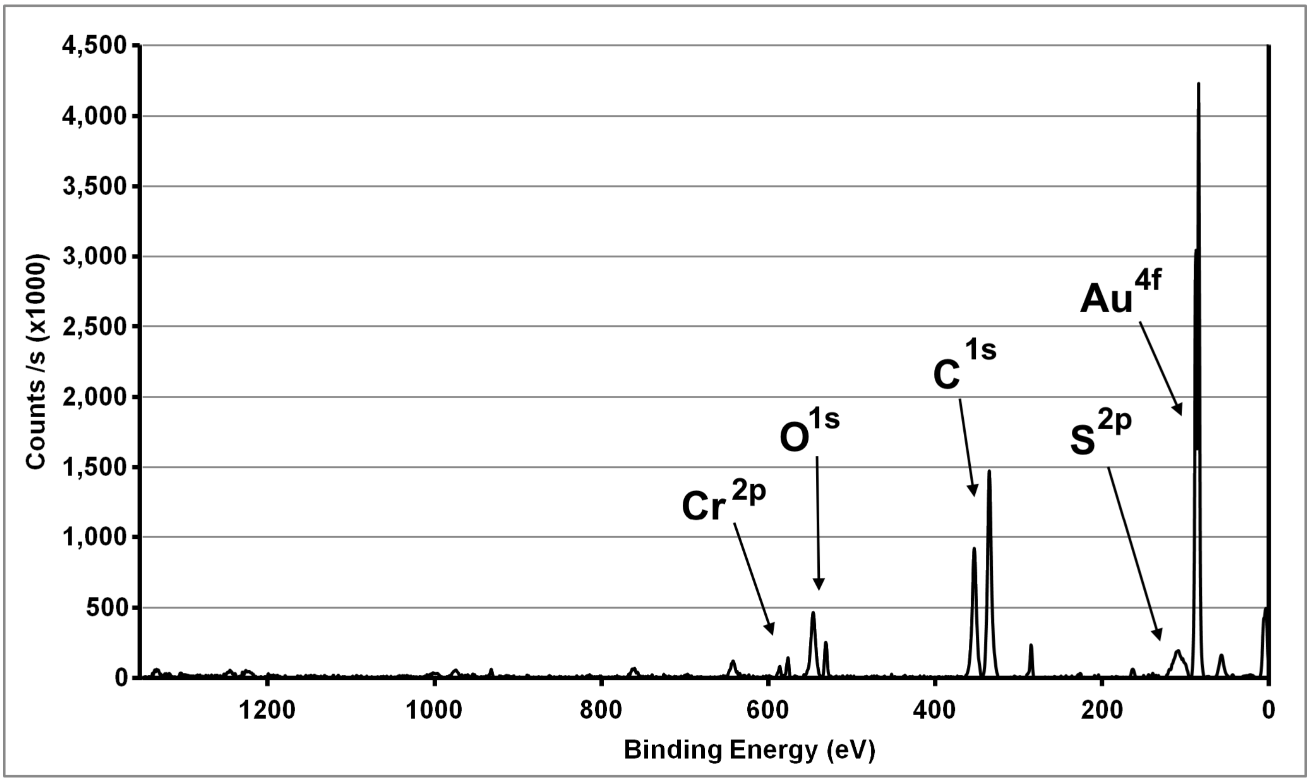

Figure S7. XPS survey spectrum of βME coated gold.

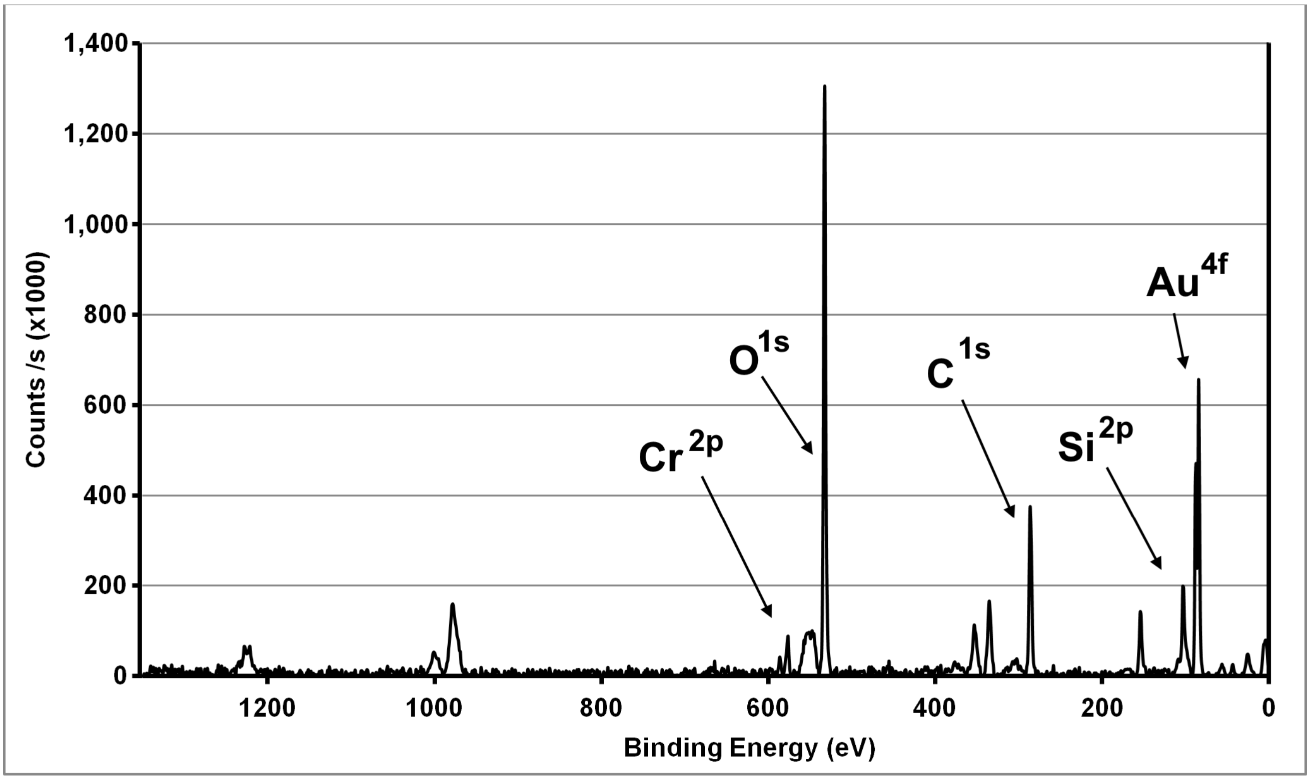

Figure S8. XPS survey spectrum of βME and Si-MEG-OH coated gold.

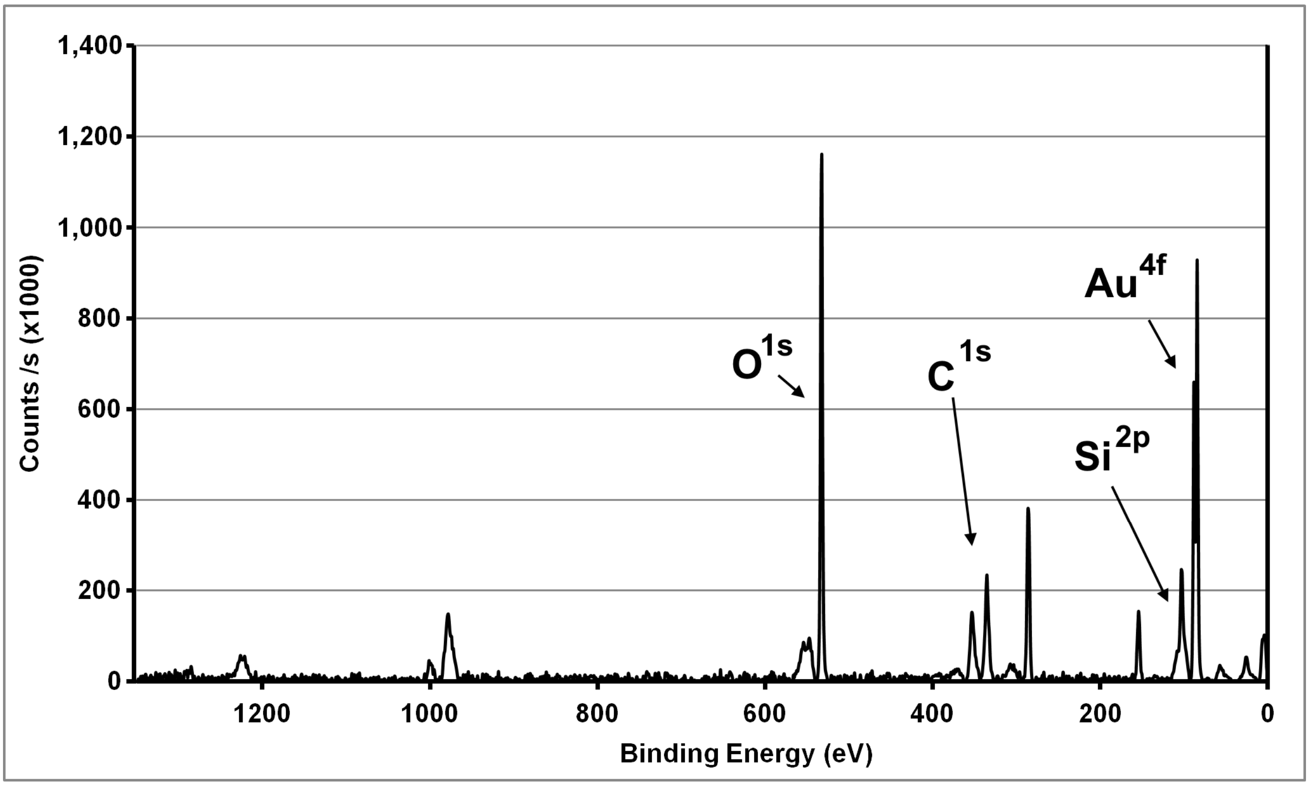

Figure S9. XPS survey spectrum of Si-MEG-OH coated gold.

S3. Supporting AFM Data

Roughness of the coatings on different substrates were calculated from the AFM data collected using Gwyddion software.

Table S1. AFM roughness data

Coating	Gold Coated Silica	Silicon	Silica Quartz Crystal
Bare	1.2	1.1	0.9
βME + Si-MEG-OH	0.1	0.2	0.3
Si-MEG-OH	0.1	0.1	0.3

Numerical values are in units of nanometers.

The profile lines of the hole in βME coating and Si-MEG-OH coating were collected from Fig. 5C using Gwyddion software.

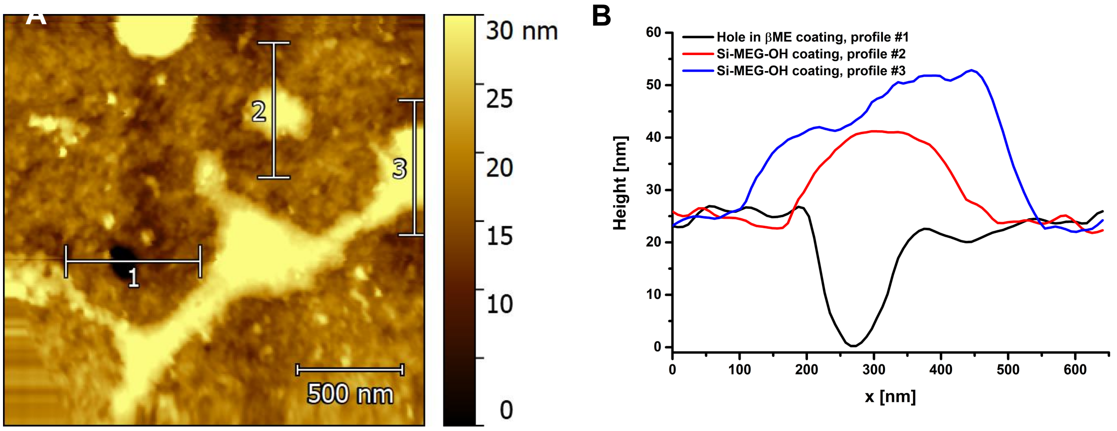

Figure S10. A. AFM image of the incomplete coating areas of the gold silicon substrate with the full βME/Si-MEG-OH coating revealing distinct layers; B. Line profiles corresponding to the hole in βME coating (1), incomplete Si-MEG-OH coating areas (2, 3).

Thus, the approximate thickness of βME coating is 20 nm, whereas for Si-MEG-OH coating it can vary from 15 to 40 nm.
